# Analysis of Metrological Quality and Mechanical Properties of Models Manufactured with Photo-Curing PolyJet Matrix Technology for Medical Applications

**DOI:** 10.3390/polym14030408

**Published:** 2022-01-20

**Authors:** Tomasz Kozior, Jerzy Bochnia, Damian Gogolewski, Paweł Zmarzły, Mateusz Rudnik, Wiktor Szot, Paweł Szczygieł, Mateusz Musiałek

**Affiliations:** Faculty of Mechatronics and Mechanical Engineering, Kielce University of Technology, 25-314 Kielce, Poland; jbochnia@tu.kielce.pl (J.B.); dgogolewski@tu.kielce.pl (D.G.); pzmarzly@tu.kielce.pl (P.Z.); mrudnik@tu.kielce.pl (M.R.); wszot@tu.kielce.pl (W.S.); pszczygiel@tu.kielce.pl (P.S.); mmusialek@tu.kielce.pl (M.M.)

**Keywords:** PJM, MED610, mechanical properties, metrological quality, 3D printing

## Abstract

This paper presents the metrological quality and mechanical properties of models in the form of hook holders manufactured from MED610 polymer material using PolyJet Matrix (PJM) technology. Measurements in the dimensional and shape analysis were made using the optical method with a microscope. The mechanical test was estimated by static tensile testing of the fabricated parts. A comprehensive approach to both the analysis of test results based on standardized samples and real hook models makes the presented results of great scientific and engineering value and creates the possibility of practical use in the medical industry, which has not been so comprehensively presented in the currently published research papers. Analyzing the results of measurements of the geometrical characteristics of the elements, it can be concluded that the PolyJet Matrix 3D printing technology has demonstrated a high level of precision in manufacturing the prototype parts. The static tensile test of samples, taking into account the printing directions, showed a high anisotropy of mechanical properties. The results of both strength and simulation tests indicate that it is necessary to assume a relatively high safety factor, the value of which depends on the direction of printing, which, in the case of such a responsible medical application, is very important.

## 1. Introduction

With the development of industry, the use of additive technologies has become increasingly common. Three-dimensional printing technologies are one of the areas that significantly contribute to the development of science, including medical sciences. Innovations in the field of medical science using 3D printing concern components and assemblies as well as the materials from which they are made. A wide range of materials, i.e., plastics, biomedical materials, ceramics and metals, are kinds of semi-finished products in 3D printing and are used for printing [1,2,3,4,5,6,7,8]. Initially, 3D printing was used for prototyping. Recently, however, 3D printing technology has gained great interest in many areas of production of components, including cylindrical ones [9,10]. It is known as additive manufacturing, which builds physical objects by applying successive layers of material of a specified thickness using a printer-understandable code generated by a so-called Slicer [11,12,13]. Compared to traditional component manufacturing methods, the ability to 3D print widens the possibilities for design, modification of a designed component, and visualization [14]. Rapid prototyping is supported by reverse engineering and has applications in the field of medical science. It enables rapid prototyping of individually adapted dimensions of a prosthesis to a patient and his/her disease. More and more often in medical applications, a combination of several technologies is used, such as 3D printing and electrospinning [15], where the PJM technology has a very large potential application [16].

The analysis of the literature and the preliminary results of our research have shown that the print direction is one of the key parameters influencing the mechanical and tribological [17] properties as well as the dimensional and shape accuracy of the models produced; therefore, in the presented work, the sample models were made in several different locations on the building platform.

The production of models from biomedical materials is accompanied by PolyJet Matrix (PJM) printers, the principle of which is as follows: the printing heads spray the material layer by layer onto the platform, which is cured by ultraviolet light [18,19,20]. This technology is very precise and applied due to the types of materials used. Prototypes should be prepared in advance, i.e., if multicolored fragments of the model are used, appropriate STL files should be prepared separately and the entire project should be exported as OBJ/VRML files [21]. The printing speed and precision depend on the choice of one of three modes, i.e., high quality, high speed and digital material. Models made with PolyJet Matrix technology use additional material for support structures. They are formed by a separate group of heads, which can be removed using pressure washers, solutions or mechanical separation using water [22,23].

This work presents a model of a medical robot’s hook holder made of MED 610 material, which, suitably adapted, could serve as part of the manipulator in the medical robot used in surgical operations for direct interaction with the human body. Its rounded working surface and design are adapted to move and hold small veins or tissues so that they are not damaged [24]. The medical robot enables not only the visualization of the surgical field in a three-dimensional image through tenfold magnification. It also enables making incisions, supporting and moving tissues and burning them. It further reduces human factors in the form of hand tremors and imprecision in some surgeons [24,25,26]. The robot’s hook holder, made of a biocompatible polymer with living tissue, appears to be more natural than a rigid handle made of stainless steel. However, it must meet the appropriate strength and geometric requirements.

New additive solutions are entering the industrial application space as 3D printing evolves. One such application is robotics, which in combination with 3D printing, is the future of intelligent robots. By designing in CAD, designers can tailor the robot to their specific needs. This provides the freedom to add new features to the models. The 3D printing allows designers to forget about the current limitations resulting from the use of conventional manufacturing methods and open up new possibilities. It allows the robot arm to be better adapted and optimized to different operational requirements [27,28]. Medical robots designed to perform medical operations usually do not work independently but support medical personnel, increasing the precision and quality of their work [29].

Based on a preliminary review of the literature, it can be concluded that additive technology is starting to become increasingly important in the medical field. The growing importance of 3D printing in medicine is accompanied by the development of biocompatible materials. Therefore, it seems reasonable to carry out research into, among other things, mechanical strength. This paper describes the results of testing the dimension and shape precision and mechanical strength in the potential application of hook-holder models intended for use in the medical robot.

## 2. Materials and Methods

### 2.1. PJM Technology

Test samples were prepared using a machine Connex 350 (Stratasys corp. Rehovot, Israel), which is the high photo-curable liquid polymer resin PolyJet Matrix (PJM). The samples were made in high quality mode. This makes it possible to make models that are impossible to produce by using conventional technologies, e.g., cell structures. The implementation of models with cellular structures in different orientations using 3D printing technology may affect the strength properties of the structures. The PolyJet Matrix technology is characterized by the continuous application of material from various groups of print heads. This process, in contrast to other technology, eliminates the need for re-exposure after the printing element. PolyJet Matrix technology allows you to work in three modes:

- High speed (HS)—with a material layer height of 32 µm, which is characterized by high-speed printing parts;
- High quality (HQ)—with a material layer height of 16 µm, which is characterized by a lower print speed and very high accuracy;
- Digital material (DM)—with a material layer height of 32 µm, which is characterized by the combination of different materials.

### 2.2. Material MED610

MED610 (Stratasys corp. Rehovot, Izrael) is a biocompatible material used in PolyJet printing technology for medical and dental applications. The manufacturer states that the material may remain permanently in contact with the skin (more than 30 days) and for a limited time in contact with the oral mucous membrane (up to 24 h). The material has been developed in accordance with accepted standards taking into account: cytotoxicity (EN ISO 10993-5:2009) [30], irritation and hypersensitivity type IV (EN ISO 10993-10:2013) [31], genotoxicity (EN ISO 10993-3:2014) [32], chemical characterization (EN ISO 10993-18:2009) [33]. The characteristics are given in Table 1 together with the standard/procedure by which they are measured and with the composition, respectively, where it is presented according to percentage weights [34,35].

### 2.3. Measurement Technologies

The measuring instruments used to measure internal and external dimensions are the MarVision MM 320 Microscope (Mitutoyo, Kawasaki, Japan) and the Mitutoyo Digital Micrometer. The Mahr microscope allows geometrical features to be measured using an integrated CCD camera. It is based on a hardened granite base together with an XY table. The CCD camera and the 0.7–4.5x Navitar Zoom lens are mounted on a stable Z column with a vertical motion of 200 mm. A digital micrometer (Mitutoyo, Kawasaki, Japan) with a measurement resolution of 0.001mm was used to measure the thickness of the A and B hook holders at four locations.

**Figure 1:**
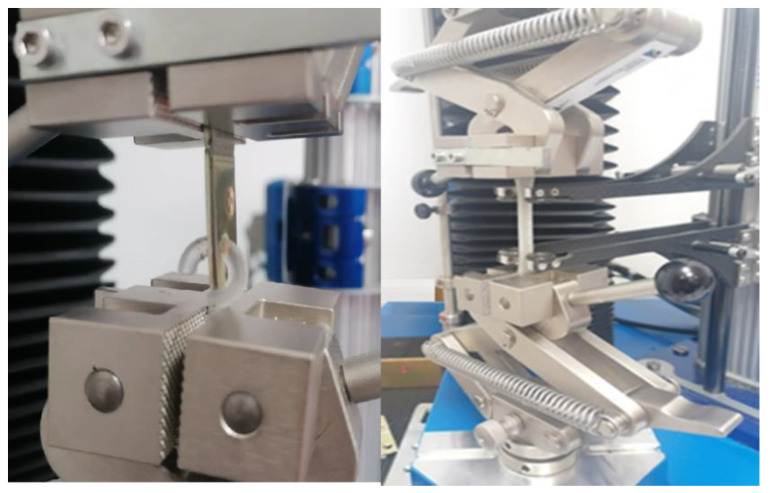
Tensile test of ISO 527 sample and hook holder.

The Inspekt Mini strength testing machine from Hegewald and Peschke MPT GmbH with the LabMaster software (Hegewald and Peschke, Nossen, Germany) was used to test strength at the maximum load of 3 kN. An example of a hook holder and tensile samples during testing is shown in Figure 1. The values of the individual quantities were calculated from the following formulas [36]:

- Mean value

(1) $$\bar{x}=\frac{\sum {}_{i=1}^{n}x_{i}}{n}$$

where: n—group size; $x_{i}$—single test result

- Standard deviation

(2) $$SD=\sqrt{\frac{1}{\left(n-1\right)}\sum {}_{i=1}^{n}{\left(x_{i}-\bar{x}\right)}^{2}}$$

where: n—group size; $x_{i}$—single test result; $\bar{x}$—test mean value;

- Sample cross-section

(3) Section=a⋅b

where: a—sample width value; b—sample thickness value

### 2.4. Samples Preparation

**Figure 2:**
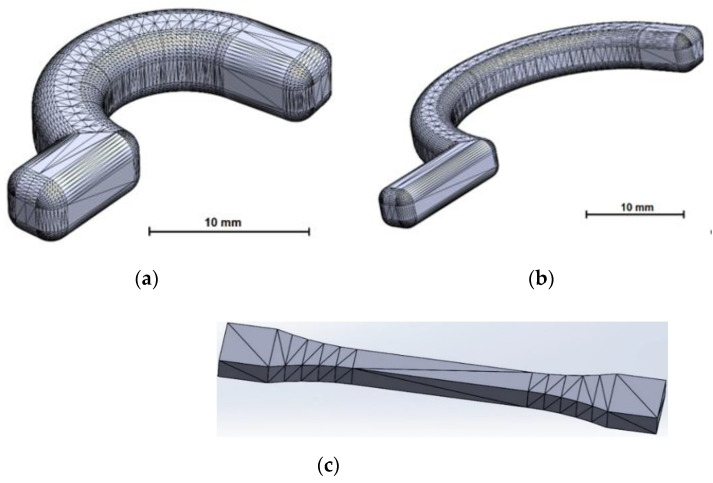
Samples: (**a**) hook holder A as an approximated triangle mesh; (**b**) hook holder B as an approximated triangle mesh; (**c**) test according to ISO 527 as an approximated triangle mesh.

The hook holders for the medical robot were designed using SolidWorks software (Dassault Systemes SolidWorks Corp., Waltham, MA, USA) and saved in STL format, which gives the solid model a mesh composed of triangles. The output for the holders is binary and the units in the file are in millimeters. In order to distinguish the hook holders, they have been given symbolic designations: A—17-mm-long hook holder; B—34-mm-long hook holder. For hook holder A, a fine resolution was adopted, which gives a linear deviation of approximately 0.009 mm and an angle of 10° (Figure 2a). For the second hook holder B, the fine resolution STL format gives a linear deviation of approximately 0.017 mm and an angle of 10° (Figure 2b).

Samples for the strength tests were designed in accordance with ISO 527 using SolidWorks software and saved in STL format with the options: deflection 0.0364 mm and angle 10° (Figure 2c).

**Figure 3:**
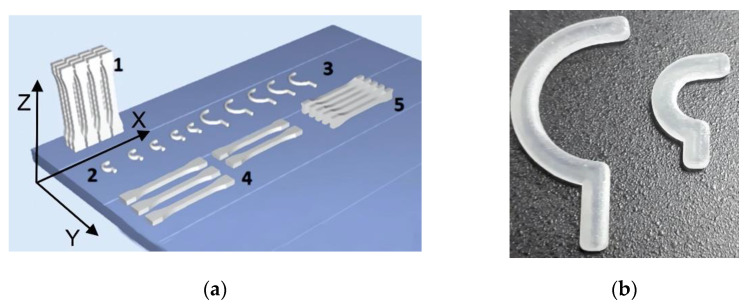
Samples: (**a**) arrangement of samples and handles on the work platform, where: (1)—vertical orientation of the sample pack—OZ direction, (2)—hook holder A, (3)—hook holder B, (4)—flat setting, (5)—side setting—OX direction; (**b**) printed hook holders.

STL files of the hook-holder models and sample were placed in Objet Studio on the virtual work of the Connex 350 printer in three characteristic directions (Figure 3a). The printout was made in high quality mode in matt mode.

The hook holders A and B (Figure 3b) are made from MED 610 (Table 1) using PJM additive technology. The print had a layer height of 0.016 mm. In order to easily distinguish between the two types of hook holders, they are marked: A—17 mm long hook holder; B—34 mm long hook holder.

## 3. Results

### 3.1. Dimensional and Shape Precision

**Figure 4:**
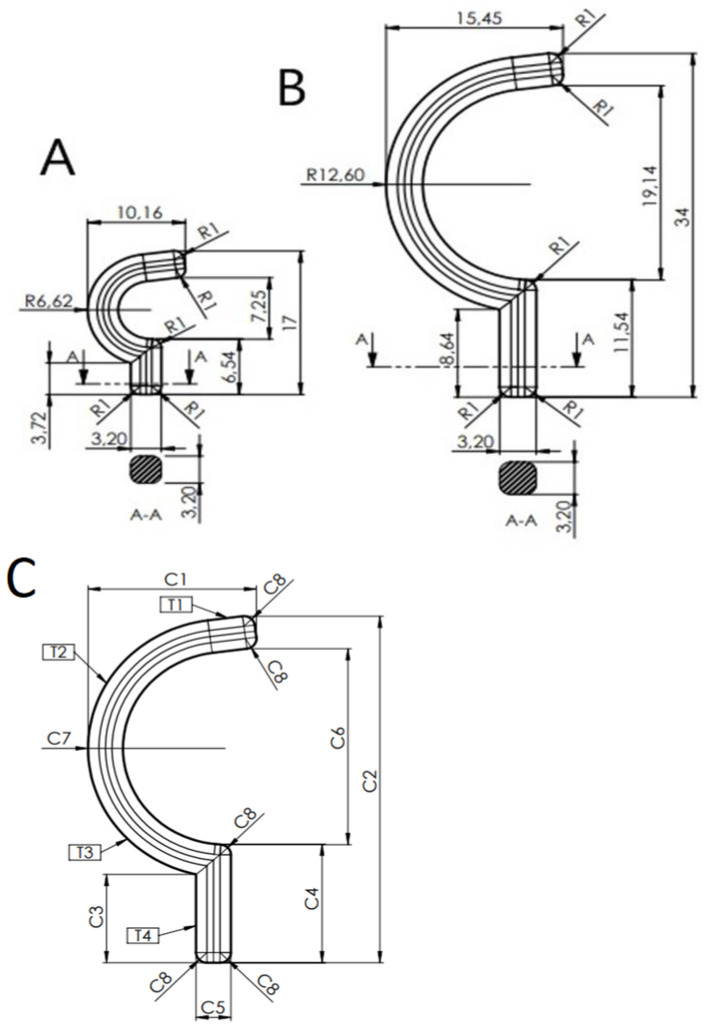
Geometrical characteristic of hook holder and CAD dimension of: (**A**) hook holder—length 17 mm, (**B**) hook holder—length 34 mm, (**C**) metrological features.

The number of samples used for measurement is five for each type of medical robot hook holder. Figure 4 shows the markings of the measured geometric features. Geometric features defining length and radii were marked C1–C8 and features defining thickness as T1-T4. Figure 4 also shows the dimensions of the hook holders A and B, which were designed using SolidWorks software. In the following analysis they will be denoted by the abbreviation CAD. Table 2 shows the results of the measurements of the A and B hook holders. In addition, basic statistics were calculated: mean value and standard deviation from formulas 1 and 2.

From Table 2, it can be seen that the standard deviation value was the lowest for T2 thickness and the highest standard deviation value was for the C2 geometric characteristic in the A hook holder. The lowest value of the standard deviation was for T2 thickness and the highest standard deviation value was for the C2 geometric characteristic in the A hook holder.

For the graphs showing the geometric values of the holders A and B (Figure 4), the following conditions were applied:

- The red line represents the CAD form, which is the value imposed by the research team to make the holder.
- The black line named, respectively, 0.1 and −0.1 mm indicate the PolyJet printing precision, i.e., 0.1 mm.
- The average CAD value columns show the difference between the mean value and the value used in the CAD drawing, i.e.,
- Difference = average value − CAD value (4).
- Where the difference between the values will be represented as a mean value. The columns show how the mean value deviates from the given CAD dimension.
- The brown, red, blue, black and orange colored points represent the geometrical characteristics C1–C8 and T1–T4 for the measured holder.

These values are the result of the difference between the value of the sample number for the relevant characteristic and the value adopted in the CAD drawing. If the columns and points take positive/negative values, it means that the characteristics deviate from the value imposed in the holder design.

### 3.2. Metrological Analysis

Table 3 and Table 4 show the geometric quality of printed elements.

The values of geometrical feature C1 indicate that only one value is around the print precision boundary, i.e., for the holder A2; however, it is 0.1 mm away from the imposed guideline. Other features are concentrated far from the set boundaries. There is also a large gap between the minimum and maximum values. A similar situation occurs for feature C2; however, for the holder A1 the value is almost in the middle of the −0.1 to 0 range. For feature C3, the four values are between −0.1 and 0.1 mm. The results of the C4 feature measurements are located above the 0.1 mm limit. Excluding the A4 holder measurement value, the rest of the value oscillates around the mean value. For the C5 feature, the values oscillate between 0 and 0.1 mm.

**Figure 5:**
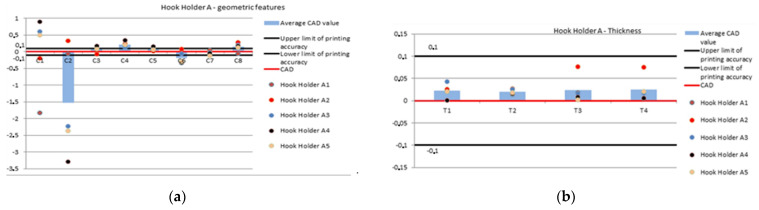
Geometric features (**a**) and thickness (**b**) of hook holder A.

Excluding the holder A4, each value is around the measurement precision. The values for feature C6 are for the holder A2 in the range 0–0.1mm and A3 in the range −0.1 mm–0. The other values of this feature oscillate around each other without exceeding the precision of PJM printing. For feature C7, it can be observed that only one of the values for the holder A5 exceeds the imposed printing precision; however, each of the other values oscillates around the CAD dimension. For feature C8, it can be observed that most of the measurement values are around the upper limit of printing precision. The exception is the A1 holder feature, which is almost in the middle of the 0.1 mm–0 range. In the case of features C1, C3 and C5, the mean value is within the limits of the part printing precision with the PJM additive technology. For features C1, C2, C6 and C7, the mean shows negative values for various reasons. In the case of feature C1, it is a single dimension that has a significant effect on the result, and in the case of features C2, C6, C7 there are negative values of dimensions that have a significant effect on the mean value. For feature C6 the values are in the 1:4 ratio, where the negative values are clustered against each other. In the case of feature C7, the mean values are 2:3 (Table 3, Figure 5).

**Figure 6:**
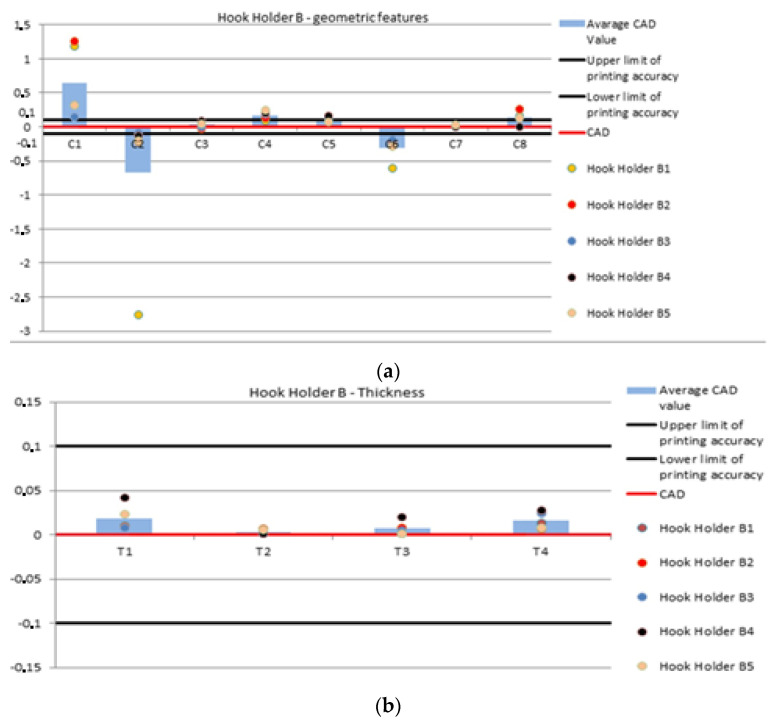
Geometric features (**a**) and thickness (**b**) of hook holder B.

For feature C1, all points are concentrated outside the printing precision limit. The closest to this limit is the feature for the B3 holder. It can also be observed that the values of this feature for the B4 and B5 holders are close to each other, while models B1 and B2 show a high deviation from the value assumed in the CAD design. For the C2 feature, values are around the lower limit of printing precision. However, only the B3 model is in the −0.1 mm–0 range. The values of B4 and B5 are outside the range, with feature C2 for model B5 being almost 0.1 mm away from the assuming limits. For feature C3, each measurement value is in the range −0.1–0.1 mm. For feature C4, only model B1 has a value within the mentioned range. The values for the models B3–B5 are around the 0.2 mm value. The C5 features for each of the hook holders are located around the upper precision limit, but only B1 and B5 are in the 0–0.1 mm range. For each of the holders, the C6 feature is smaller than the assumed CAD model, and none of them are in the assumed range. The values of feature C7 compared to the others are furthest from the assumed limits and closest to the values assumed in the CAD model. For feature C8, the closest to the assumed value in the CAD model is the feature in part B4. The feature values for the other details are outside the designated range. The average deviations from the CAD values take a negative value only twice. For feature C2, the value for part B1 plays a significant role. A different situation occurs with the C6 feature, where it can be observed that the dimensions are smaller than assumed (Table 3, Figure 6).

The thickness of the A1–A5 holders is not beyond the limits of 3D printer precision. For each of the thicknesses, the values are higher than designed and are within the range of 0–0.1 mm. The values are divergent, but the mean value of each space on the handle is similar (Table 4, Figure 5).

The thickness for the holders B1–B5 shows greater precision than for the holders A1–A5. The largest deviation is the T1 measurement value for the B4 holder. The smallest deviations from the CAD value occur sequentially for the features: for T1, the smallest deviation occurs with model B3. The feature T2 shows the lowest value in the models B1 and B4. At feature T3, model B5 shows the highest precision, and at T4, model B2 (Table 4, Figure 6).

This section may be divided by subheadings. It should provide a concise and precise description of the experimental results, their interpretation, and the experimental conclusions that can be drawn.

### 3.3. Mechanical Strength

The static tensile test of the hook holders of the medical robot was performed using an Inspekt Mini strength testing machine with Labmaster software. Tests were performed on five pieces of holders of each type: A and B. The values of the maximum tensile force were determined. The results of the tensile tests on the hook holder models are shown in Table 5.

From Table 5, it can be observed that the mean value of the maximum load force for the hook holder B is 1.85% greater than the mean value of the maximum force for the hook holder A.

The standard deviation values for the measured maximum force for both types of hook holders can be considered quite large. For the holder A, the ratio of the standard deviation to the mean value of the maximum force was 21.8%, while for the holder B it was 17.4%. This means that the measured values are very far from the mean value. The standard deviation of the measured maximum force for the hook holder A was 22.67% greater than the standard deviation for the hook holder B.

**Figure 7:**
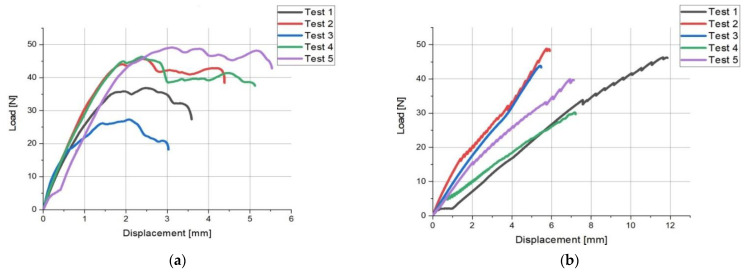
Tensile charts for: (**a**) hook holder A, (**b**) hook holder B.

Figure 7 shows the tensile curves of the load force–elongation (load-displacement) relationship for the hook holders A and B.

Bursting of the hook holders occurred at elongation: in the range from 1 to 5.5 mm for the holders A (Figure 7a); in the range from 4.5 to 12 mm for the holders B (Figure 7b).

Table 6 shows the tensile results of the samples. The table includes the test number, the cross-section calculated from formula 4, the maximum force, the tensile strength and the printing direction of the samples.

By analyzing Table 6, it can be observed that the largest cross-section was that of the sample printed in the direction of the OZ axis: 21.3725 mm^2^. The sample printed in the OY direction had the smallest cross-section: 19.9796 mm^2^.

The highest mean maximum force occurred for the sample printed in the direction of the OX axis: 1047.08 N. In contrast, the lowest mean maximum force was achieved by the sample printed in the OZ direction: 342 N. The highest mean maximum force was 206.13% greater than the lowest mean maximum force. The mean value of the maximum force of the printed sample in the direction of the OY axis was 189.01% greater than the lowest value of the maximum force. However, the value of the mean maximum force of the sample printed in the OX direction was 5.94% higher than the mean maximum force for the sample printed in the OY direction.

The highest mean tensile strength value occurred for the sample printed in the OX direction and was 226.01% higher than the lowest mean tensile strength value found for the OZ sample. The mean tensile strength of the printed sample in the OY direction was 209.16% higher than the lowest value of the mean tensile strength.

The mean tensile strength of the sample printed in the OX direction was 5.45% higher than the mean tensile strength of the sample printed in the OY direction.

The ratio of the standard deviation to the mean of the maximum tensile force was:

- For the printing direction OX—1.29%,
- For the printing direction OY—2.32%,
- For the printing direction OZ—9.84%.

The ratio of the standard deviation to the mean tensile strength was, in percentage terms, the same as the ratio of the standard deviation to the mean maximum tensile strength, which is obvious. It is clear that the greatest dispersion of results of both maximum tensile force and tensile strength occurred for samples printed in the direction of OZ, that is, in the vertical direction.

**Figure 8:**
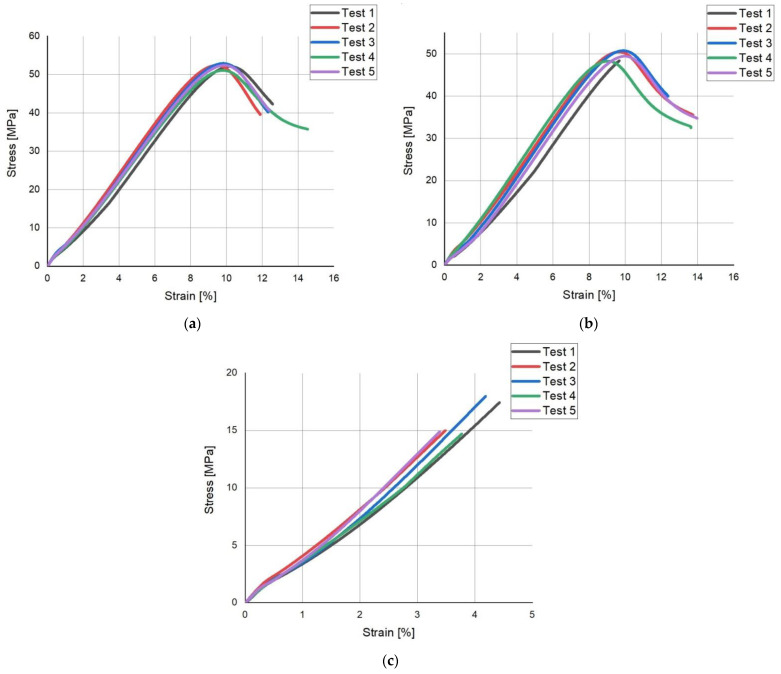
Samples axis: (**a**) OX, (**b**) OY, (**c**) OZ.

Figure 8 shows the tensile-strain curves of ISO 527 samples. Based on the charts (Figure 8), a small dispersion of test results can be observed. It is also worth noting that the samples printed in the OX direction reached the values of the highest stress according to the chart (Figure 8a) in the range: (51–54) MPa at a strain of (9–10)%. Samples printed in the OY direction reached values of the highest stress according to the chart (Figure 8b) in the range: (45; 52) MPa for strain in the range: (8.8; 10)%. For samples printed in the OZ axis, the highest stress values according to the chart (Figure 8c) were in the range: (14–18) MPa at a strain of (3.5; 4.5)%.

The breaking of the samples occurred in ranges of strain percentages:

- OX in the range (11.9; 14.5)% Figure 8a,
- OY in the range (9.8; 14)% Figure 8b,
- OZ in the range (3.3; 4.5)% Figure 8c.

### 3.4. Solidworks Simulation

**Figure 9:**
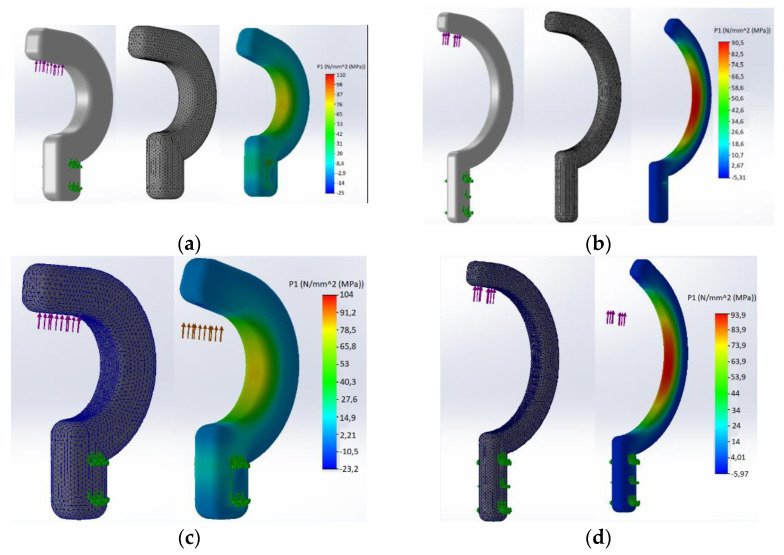
The mesh used, the load and the simulation result for: (**a**) hook holder A—mesh not densified, (**b**) hook holder B—mesh not densified, (**c**) hook holder A—mesh densified, (**d**) hook holder B—mesh densified.

The simulation was performed to show the critical areas of the hook holder structure where the highest stresses due to tensile force occur. The results were calculated assuming a viscoelastic body model for the material data in Table 1. The maximum force was 40 N. Two types of simulations were performed for both hooks, where the changed test parameter was the size of the mesh. Figure 9a,b shows the simulation results for models with a mesh with the same size as a single element, respectively, 0.301 and 0.377 mm. In the case of the models presented in Figure 9c,d, the mesh retained the previous values of a single element, but at the place of its internal radius, its density was applied, where individual elements in these places were characterized by the following sizes: small hook −0.135 mm and large hook 0.169 mm. The type of mesh, the method of applying the external force and the result of the simulation calculations for the B and A hook holders are shown in Figure 9. The parameters of the generated meshes for both types of samples are presented in Table 7.

From the simulations presented, it appears that the concentration of stresses occurs on the inner part of the arc of the hook holder. For mesh (not densified), the A-hooks were about 70 MPa, and for the B-hooks, 90.5 MPa. For mesh (densified), the A-hooks were about 80 MPa and, for B-hooks, 93.9 MPa. Stresses are local in nature, causing the material to lose local cohesion, which progresses gradually into the material leading to the final break, which is also confirmed by the shape of the tensile charts of the individual hook holder models (Figure 9).

## 4. Discussion

Analyzing the results of the hook holder measurements (Table 2), it can be concluded that the highest value of standard deviation occurs for the geometrical feature C2 (hook holder length) in both types of the hook holder. The thickness measurement showed that the values obtained were within the manufacturing precision limits of the 3D printer used (Table 4). This has been achieved thanks to a very precise saving of STL files in a customized mode with high resolution and a triangulation process.

From the results of the static tensile tests performed on the hook holders (Table 5), it was observed that the value of the mean maximum load force on hook holder B is 1.67% higher than the value of the maximum load force on hook holder A, so this is not a significant difference. Analyzing the results of measurements of selected geometrical features of the samples printed in three directions (Table 6), it was found that the largest cross-section had the sample printed in the OZ direction (21.37 mm^2^) and the smallest in the OY direction (19.98 mm^2^). Static tensile testing of samples printed in three directions (Table 6) showed that samples printed in the OX direction had the highest value $\bar{R_{m}}$ and this was 226.01% higher than the lowest value $\bar{R_{m}}$ in samples printed in the OZ direction. The results of the research confirm the previously conducted literature research, where the differences in the strength of the models produced for different orientations on the building platform were clearly indicated. However, the calculated differences are so large that they require taking into account additional safety factors for selected print variants, which significantly complicates the design and production of hook-type elements for medical/surgery application, which often work under load in several different axes. For samples printed in the OY direction, the value $\bar{R_{m}}\;$ was 209.16% higher than $\bar{R_{m}}$ samples printed in the OZ direction. The mechanical properties specified by the producer for MED610 material are presented for the material before printing. Therefore, for the OX axis, the values fluctuate in the range presented by the producer. In the case of samples printed on the OY axis, the values fluctuate around the lower values specified by the producer, i.e., 50 MPa. However, in the case of the OZ axis, the values are lower than those provided by the producer by 71%. Based on the strength analysis performed in Solidworks (Figure 9), it was found that the stress concentration occurs at the largest radius of the hook holder—feature C7 (Figure 4). The highest local stress (mesh not densified) for the hook holder B was 90.5 MPa, while for the hook holder A it was 70 MPa. The difference is significant, the maximum stresses in the hook holder B being about 22% higher than those in the hook holder A. On the other hand, in the case of the mesh with increased local density, hook A and hook B were characterized by maximum stresses in the radius area equal to 80 and 93.9 MPa, respectively. In this case, the difference was 17%. The explanation of it is the fact that crack initiation does not only require a certain level of stress to be reached but also needs to fulfill an energy criterion and thus allows for local stresses larger than the material tensile strength; this phenomenon was fully described in the paper [37]. Such differences indicate that in the case of designing elements for the medical industry made of MED 610 material, additional corrections should be made in sensitive points, such as ribs or thickenings to prevent failures and increase the reliability of the device.

## 5. Conclusions

Based on the research results, it is possible to formulate the following general conclusions:

The dimension and shape precision analysis showed highly satisfactory precision, which is sufficient for applications in the medical industry.

Static tensile testing of samples printed in three directions showed an anisotropy of the mechanical properties of the material tested. The mean tensile strength in the OZ direction was 16 MPa, while in the OX direction it was 52.01 MPa, where the difference is equal to more than 200%. This means that when designing this type of component, an appropriate safety factor must be adopted to reduce the allowable force transmitted by the hook holder, which will be manufactured using 3D printing technology.

The printed bio-element from MED 610 does not have electromagnetic properties, which is definitely an advantage, allowing the hook holder to work in the vicinity of heart or other organs that are susceptible to electromagnetic effects. A hook holder made with 3D printing technology can have any shape while retaining medical properties. Using biomedical materials, the Connex 350 printer is ideal for use in this field. The analysis of the obtained results allowed for the development of a further series of new designs to meet the requirements of modern materials more effectively, i.e., biodegradability, biotolerance and biofunctionality.

The optimum selection of printing parameters is necessary and important for the durability and functionality of the model. These parameters should be adjusted by the model designer, as only the model designer knows its purpose and functions.

## Figures and Tables

**Table 1:** Properties and chemical composition of MED 610 material [34,35].

	Properties		
Property	Standard		Value
Tensile strength	D-638-03		50–65 MPa
Ultimate elongation	D-638-05		10–25%
Young’s modulus	D-638-04		2000–3000 MPa
Bending strength	D-790-03		75–110 MPa
Modulus of elasticity in bending	D-790-04		2200–3200 MPa
Poisson ratio *	ASTM D638-10		0.41
Deflection temperature (under load of 0.46 MPa)	D-648-06		45–50 °C
Water absorption	D-570-98 24HR		1.1–1.5%
Shore hardness	D Scale		83–85 D
Rockwell hardness	M Scale		73–76 M
Biocompatibility	PN-EN ISO 10993-1:2017		Skin contact: - more than 30 days Contact with mucous membrane: - up to 24 h
	**Chemical composition**		
**Component**		**% of weights**	
Isbornyl acrylate		15–30	
Acrylic monomer		15–30	
Urethane acrylate		10–30	
Acrylic monomer		5–10; 10–15	
Epoxy acrylate		5–10; 10–15	
Arylate oligomer		5–10; 10–15	
Photoinitiator		0.1–1; 1–2	

* Note: the Poisson ratio value was obtained by e-mail from Stratasys at the authors’ request.

**Table 2:** Results of measurements of geometrical features, values in mm.

Geometric Characteristic and Thickness (mm)	CAD Dimensional	Samples					Average	SD	Hook Holder
		1	2	3	4	5			
**C1**	10.16	8.333	9.956	10.767	11.052	10.663	10.1542	1.095	A
**C2**	17	16.945	17.325	14.766	13.706	14.637	15.476	1.575	
**C3**	3.72	3.795	3.642	3.799	3.893	3.807	3.787	0.091	
**C4**	6.54	6.684	6.773	6.671	6.883	6.737	6.75	0.085	
**C5**	3.2	3.305	3.223	3.302	3.356	3.26	3.289	0.05	
**C6**	7.25	6.912	7.33	7.193	6.924	6.966	7.065	0.187	
**C7**	6.62	6.666	6.642	6.607	6.580	6.494	6.598	0.066	
**C8**	1	0.949	1.279	1.205	1.1741	1.125	1.147	0.111	
**T1**	3.2	3.221	3.226	3.243	3.201	3.221	3.222	0.015	
**T2**	3.2	3.215	3.227	3.226	3.216	3.218	3.220	0.006	
**T3**	3.2	3.218	3.276	3.216	3.208	3.202	3.224	0.03	
**T4**	3.2	3.205	3.275	3.217	3.206	3.221	3.225	0.029	
**C1**	15.45	16.631	16.709	15.601	15.775	15.771	16.097	0.528	B
**C2**	34	31.241	33.79	33.921	33.876	33.804	33.326	1.167	
**C3**	8.64	8.701	8.616	8.646	8.733	8.679	8.675	0.046	
**C4**	11.54	11.627	11.665	11.734	11.74	11.79	11.711	0.067	
**C5**	3.2	3.298	3.361	3.35	3.365	3.28	3.331	0.039	
**C6**	19.14	18.529	18.93	18.955	18.87	18.856	18.828	0.528	
**C7**	12.6	12.595	12.628	12.626	12.608	12.628	12.617	0.172	
**C8**	1	1.159	1.255	1.129	0.998	1.125	1.133	0.092	
**T1**	3.2	3.21	3.209	3.208	3.242	3.223	3.218	0.015	
**T2**	3.2	3.201	3.207	3.203	3.201	3.206	3.204	0.003	
**T3**	3.2	3.202	3.208	3.205	3.22	3.201	3.207	0.008	
**T4**	3.2	3.214	3.207	3.224	3.228	3.208	3.216	0.009	

**Table 3:** Geometric features of hook holder A and B.

**Hook Holder A**								
	**C1** **(mm)**	**C2** **(mm)**	**C3** **(mm)**	**C4** **(mm)**	**C5** **(mm)**	**C6** **(mm)**	**C7** **(mm)**	**C8** **(mm)**
**A1**	−1.827	−0.055	0.075	0.144	0.105	−0.338	0.046	−0.051
**A2**	−0.204	0.325	−0.078	0.233	0.023	0.08	0.022	0.279
**A3**	0.607	−2.234	0.079	0.131	0.102	−0.057	−0.013	0.205
**A4**	0.892	−3.294	0.173	0.343	0.156	−0.326	−0.04	0.174
**A5**	0.503	−2.363	0.087	0.197	0.06	−0.284	−0.126	0.125
**Difference (mean value)**	−0.006	−1.524	0.067	0.21	0.089	−0.185	−0.022	0.147
**Hook Holder B**								
	**C1** **(mm)**	**C2** **(mm)**	**C3** **(mm)**	**C4** **(mm)**	**C5** **(mm)**	**C6** **(mm)**	**C7** **(mm)**	**C8** **(mm)**
**B1**	1.181	−2.759	0.061	0.087	0.098	−0.611	−0.005	0.159
**B2**	1.259	−0.21	−0.024	0.125	0.161	−0.21	0.028	0.255
**B3**	0.151	−0.079	0.006	0.194	0.15	−0.185	0.026	0.129
**B4**	0.325	−0.124	0.093	0.2	0.165	−0.27	0.0078	−0.002
**B5**	0.321	−0.196	0.039	0.25	0.08	−0.284	0.028	0.125
**Difference (mean value)**	0.647	−0.674	0.035	0.171	0.131	−0.312	0.017	0.133

**Table 4:** Thickness of hook holder A and B.

**Hook Holder A**				
	**T1** **(mm)**	**T2** **(mm)**	**T3** **(mm)**	**T4** **(mm)**
**A1**	0.021	0.015	0.018	0.005
**A2**	0.026	0.027	0.076	0.075
**A3**	0.043	0.026	0.016	0.017
**A4**	0.001	0.016	0.008	0.006
**A5**	0.021	0.018	0.002	0.021
**Difference (mean value)**	0.023	0.02	0.024	0.025
**Hook Holder B**				
	**T1** **(mm)**	**T2** **(mm)**	**T3** **(mm)**	**T4** **(mm)**
**B1**	0.01	0.001	0.002	0.014
**B2**	0.009	0.007	0.008	0.007
**B3**	0.008	0.003	0.005	0.024
**B4**	0.042	0.001	0.02	0.028
**B5**	0.023	0.006	0.001	0.008
**Difference (mean value)**	0.018	0.004	0.007	0.016

**Table 5:** Test results of hook holder.

Test Number	Cross-Section (mm)	Maximum Load $F_{m_{i}}\;(N)$	Hook Holder
1	10.24	36.8	**A**
2		45.7	
3		27.3	
4		46.3	
5		49.1	
$\bar{x}$		**41.1**	
SD		**8.95**	
1	**10.24**	46.3	**B**
2		48.9	
3		43.8	
4		30.2	
5		40.0	
$\bar{x}$		**41.8**	
SD		**7.3**	

**Table 6:** Tensile test results of samples ISO 527.

Test Number	Cross Section of Sample S(mm^2^)	$\mathrm{Maximum}\;\mathrm{Strength}\;F_{m_{i}}\;(N)$	$\mathrm{Tensile}\;\mathrm{Strength}\;R_{m_{i}}\;(\mathrm{MPa})$	Axis
1	20.0694	1045.7	52.11	**OX**
2		1051.7	52.40	
3		1061.4	52.89	
4		1025.2	51.08	
5		1051.4	52.39	
$\bar{x}$		**1047.08**	**52.17**	
SD		**13.48**	**0.67**	
1	19.9796	967.0	48.40	**OY**
2		1008.5	50.48	
3		1014.3	50.77	
4		964.6	48.28	
5		988.2	49.46	
$\bar{x}$		**988.51**	**49.48**	
SD		**22.90**	**1.15**	
1	21.3725	372.8	17.4	**OZ**
2		320.3	15.0	
3		384.4	18.0	
4		314.3	14.7	
5		318.4	14.9	
$\bar{x}$		**342.04**	**16.00**	
SD		**33.68**	**1.58**	

**Table 7:** Mesh parameters.

	Hook A (Numbers of Nodes)	Hook A (Numbers of Triangles)	Hook B (Numbers of Nodes)	Hook B (Numbers of Triangles)
**Mesh (not densified)**	75,912	52,073	106,484	73,162
**Mesh densified**	107,395	74,441	150,692	104,287
